# Magnetic Resonance Imaging Features of Cerebral Infarction in Critical Patients Based on Convolutional Neural Network

**DOI:** 10.1155/2021/4095433

**Published:** 2021-07-26

**Authors:** Yi Bo, Junli Xie, Jianguo Zhou, Shikun Li, Yuezhan Zhang, Zhenjiang Zhou

**Affiliations:** ^1^Department of Intensive Care Unit, Lianyungang Hospital of Traditional Chinese Medicine, Lianyungang 222000, Jiangsu, China; ^2^Department of Imaging, Lianyungang Hospital of Traditional Chinese Medicine, Lianyungang 222000, Jiangsu, China; ^3^Department of Geriatric Diseases, Lianyungang Hospital of Traditional Chinese Medicine, Lianyungang 222000, Jiangsu, China

## Abstract

The clinical application of the artificial intelligence-assisted system in imaging was investigated by analyzing the magnetic resonance imaging (MRI) influence characteristics of cerebral infarction in critically ill patients based on the convolutional neural network (CNN). Fifty patients with cerebral infarction were enrolled and examined by MRI. Besides, a CNN artificial intelligence system was established for learning and training. The features were extracted from the MRI image results of the patients, and then, the data were calculated by computer technology. The gray-level cooccurrence matrix (GLCM) of T1-weighted images was 0.872 ± 0.069; the reasonable prediction (ALL) result was 0.766 ± 0.112; the gray-level run-length matrix (GLRLM) was 0.812 ± 0.101; the multigray-level area size matrix (MGLSZM) result was 0.713 ± 0.104; and the result of gray-scale area size matrix (GLSZM) was 0.598 ± 0.099. The GLCM, ALL, GLRLM, MGLSZM, and GLSZM of enhanced T1-weighted images were 0.710 ± 0.169, 0.742 ± 0.099, 0.778 ± 0.096, 0.801 ± 0.104, and 0.598 ± 0.099, respectively. The GLCM, ALL, GLRLM, MGLSZM, and GLSZM of T2-weighted images were 0.780 ± 0.096, 0.798 ± 0.087, 0.888 ± 0.086, 0.768 ± 0.112, and 0.767 ± 0.100, respectively. In short, the image diagnosis method that could reduce the subjective visual judgment error to a certain extent was found by analyzing the characteristics of MRI images of critically ill patients with cerebral infarction based on CNN.

## 1. Introduction

There are many causes of cerebral infarction in patients, but the pathogenesis is almost all due to atherosclerosis in the patient. Moreover, this atherosclerosis is a disease of the blood vessel wall itself [1]. This disease is usually accompanied by high blood pressure, diabetes, and other complications. Cerebral infarction, in pathophysiology, is the local ischemic necrosis of brain tissue resulting from further atherosclerosis. The various factors that cause a cerebral infarction, such as high blood pressure, smoking, diet, lack of exercise, excessive drinking, and even mental stress and depression, may cause cerebral infarction. Cerebral infarction has almost no prodromal symptoms at the time of onset, and the onset is acute. Besides, it usually occurs when the patient is sleeping or resting, so it is difficult to be detected by the patient or the patient's family members in time. There are usually some basic medical examinations, such as electrocardiogram and chest radiographs in the post-onset examination of cerebral infarction, but these basic examinations are only employed to detect the basic physical state of the patient, and ultimately the local brain structure imaging examination is used to assist clinical diagnosis and treatment. Imaging examination of brain structure usually refers to computed tomography (CT), X-ray, or head magnetic resonance imaging (MRI) [2]. MRI images have high resolution for soft tissues and will not cause trauma. Compared with CT, it has a higher diagnostic sensitivity and specificity and can display the three-dimensional structure of the brainstem in all directions, which helps doctors early judge acute cerebral infarction. The research content and index judgment of this study were mainly based on MRI technology.

At present, the main clinical screening methods for cerebral infarction are derived from imaging examinations. However, the human brain structure is complex and the cerebral infarction area is different, so it is not distinguished easily. MRI is relatively clearer, but it is also not easy to distinguish simply by visual discrimination [3]. In this study, it was hoped that the artificial intelligence-assisted diagnosis could help clinically better judge the patient's condition more clearly. MRI based on the convolutional neural network (CNN) can better display the image differences that cannot be distinguished by human eyes and can provide better diagnosis and treatment means for doctors in clinical treatment. CNN has made great achievements in processing medical images to assist doctors in disease diagnosis of patients. Besides, X-rays based on CNN can segment bone structure images very well, and this neural model has achieved excellent performance in three error indexes [4]. In clinical practice, it is difficult to distinguish the subtle differences of various cerebral infarction patients by simple visual judgment. By extracting certain characteristic information, an artificial intelligence system is constructed to assist the diagnosis and treatment of cerebral infarction in clinical practice.

To sum up, the application of MRI images based on CNN in clinical diagnosis of cerebral infarction patients was investigated in this study, the in-depth supervision mechanism was innovatively introduced, and the recognition ability of U-Net was enhanced, which was conducive to the accurate extraction and reconstruction of MRI images.

## 2. Methods

### 2.1. Research Objects

In this study, 50 patients with cerebral infarction were selected randomly, who were admitted to our hospital from January 2019 to January 2021. The study was approved by the ethics committee of our hospital, and the patients and their family members included in the study knew and signed the informed consent forms. Among them, 38 were males and 12 were females, with an average age of 68.98 ± 15.43 years old. The criteria for inclusion were defined to include the following: patients who had speech disorder, hemiplegia of the limbs, and movement disorders on admission, which could be preliminarily considered as cerebral infarction; had clinical symptoms and signs of acute stroke; had complete clinical data, including detailed medical records, laboratory examination indexes, imaging examination data, and pathophysiological examination data. The criteria for exclusion were defined to include the following: patients who were not diagnosed with cerebral infarction after preliminary examination, suffered from mental illness, and did not sign the informed consent form or withdrew from the experiment halfway.

### 2.2. Experimental Equipment and Operation

GE discovery750 3.0T magnetic resonance scanner was used for multisequence scanning. The scanning parameters were as follows: 8 ms and 535 ms T1-weighted imaging, 9 ms and 650 ms contrast-enhanced T1-weighted imaging, 105 ms and 3,500 ms T2-weighted imaging, 15° flip angle, 220 × 220 mm^2^ field of view, 3 mm layer thickness, and 0.9 mm layer spacing. Enhancer applied in this study was 0.5 mmol/kg gadopentetate dimeglumine (Gd-DTPA), the total dose was 20–25 mL, and there was the intravenous injection with a high pressure syringe, with a rate of 3 mL/s. The image analysis was jointly read by two experienced imaging physicians. In addition, the operator should be not aware of the clinical data of the patients examined by the MRI scan in advance.

### 2.3. Algorithm Construction

Based on the commonly used deep learning CNN, the upgraded algorithm was integrated with the multiscale framework, dense void convolutional network, attention mechanism, spatial pyramid, and other technologies by improving the design of network encoder, center, and decoder module of U-Net algorithm framework, which could be named as dense-pyramid-attention U-Net (DPA-UNet). DPA-UNet was used for intelligent recognition and accurate segmentation of patients' image, examination images, thereby accelerating the speed of convolution processing of image feature information. After multistep image optimization processing, the image quality was finally promoted and the diagnosis efficiency of cerebral infarction was improved.

As a kind of CNN, the U-Net network model also had the problem of gradient disappearance with the deepening of the network and pooling operation. The disappearance of the gradient would make the loss backpropagation invalid in the shallow network, which would inevitably slow down the convergence speed and reduce the discriminative ability of the model. To deal with this problem, the choice was made to add additional monitoring in the hidden layer of the U-Net expansion path to counter the adverse effects of gradient disappearance.

### 2.4. Evaluation Parameters

After learning and training, the CNN system was adopted to extract data such as pixel gray-level statistics of images, regional trait description, and gradient analysis of local areas. Then, the calculation was performed by computer, and the equations were as follows.

Mean value (*j*_mean) is as follows:

(1) $$\begin{array}{c} \bar{y}=\frac{1}{m}\sum_{j=1}^{m}y_{j}. \end{array}$$

Variance (*j*_std) is as follows:

(2) $$\begin{array}{c} \sqrt{\frac{1}{m}\sum_{j=1}^{m}{\left(y_{j}-y\right)}^{2}}. \end{array}$$

Degree of skewness (*j*_skewness) is as follows:

(3) $$\begin{array}{c} \frac{\left(1/m\right)\sum_{j=1}^{m}{\left(y_{j}-y\right)}^{3}}{{\left(\sqrt{\left(1/m\right)\sum_{j=1}^{m}{\left(y_{i}-y\right)}^{2}}\right)}^{3}}. \end{array}$$

Kurtosis (*j*_kurtosis) is as follows:

(4) $$\begin{array}{c} \frac{\left(1/m\right)\sum_{j=1}^{m}{\left(y_{j}-y\right)}^{4}}{{\left(\left(1/m\right)\sum_{j=1}^{m}{\left(y_{i}-y\right)}^{2}\right)}^{2}}-3. \end{array}$$

Area (*s*_area) is as follows:

(5) $$\begin{array}{c} A=\left|x\right|. \end{array}$$

Perimeter (*s*_perimeter) is as follows:

(6) $$\begin{array}{c} L=\left|y\right|. \end{array}$$

Circularity coefficient (*s*_circularity) is as follows:

(7) $$\begin{array}{c} 4\pi \frac{A}{L^{2}}. \end{array}$$

Elongation (*s*_elongation) is as follows:

(8) $$\begin{array}{c} E=\frac{n}{N}. \end{array}$$

Form factor (*s*_form) is as follows:

(9) $$\begin{array}{c} \frac{L\times E}{8A}. \end{array}$$

Contrast (*t*_contrast) is as follows:

(10) $$\begin{array}{c} \sum_{j}\sum_{k}{\left(j-k\right)}^{2}L\left(j,k\right). \end{array}$$

Correlation (*t*_correlation) is as follows:

(11) $$\begin{array}{c} \frac{\sum_{j}\sum_{k}\left(jk\right)L\left(j,k\right)-\varphi_{y}\varphi_{z}}{\lambda_{y}\lambda_{z}}. \end{array}$$

Entropy (*t*_entropy) is as follows:

(12) $$\begin{array}{c} -\sum_{j=1}^{p}\sum_{k=1}^{p}L\left(j,k\right)\log\left(L\left(j.k\right)\right). \end{array}$$

An ellipse surrounding the mass could be formed by the smallest axis *n* and the largest axis *N*; *L* (*j*, *k*) is the gray-level probability of *j* and *k* in the gray-level cooccurrence matrix (GLCM); *y* and *z* are the partial probability density functions; *L* is the gray-level quantization level; *n* is the number of pixels in the region of interest; *yj* is the gray-level value of the *j*-th pixel.

GLCM refers to a common method to describe the texture by studying the spatial correlation characteristics of gray. The gray-level run-length matrix (GLRLM) is applied to describe the distribution of pixel values. The gray-level size zone matrix (GLSZM) is adopted to describe the probability density of the conditional distribution of two variables of image brightness. Besides, the reasonable prediction (ALL) is the estimated value of the usual situation.

**Figure 1:**
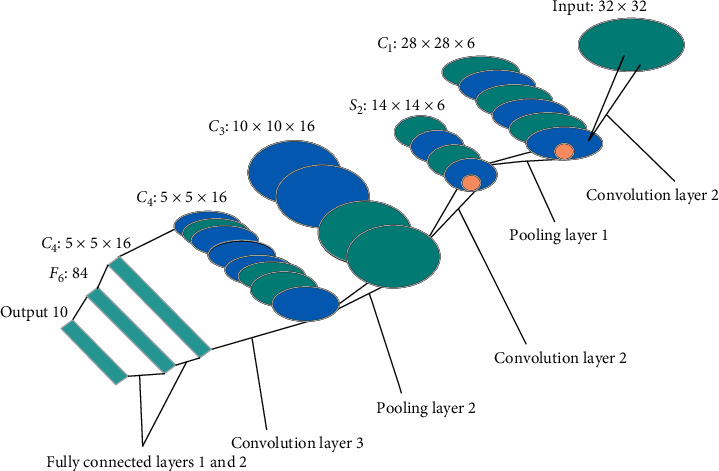
Flow chart of classic CNN.

It should be noted that, for each image case of MRI sequence, when different feature descriptions were used for auxiliary diagnosis, the system would be randomly trained and tested for 100 times, so as to obtain the average value of the final measurement parameters. This average value could fully express the comprehensive performance of the current artificial intelligence assisted diagnosis system (Figure 1).

### 2.5. Statistical Methods

SPSS22.0 statistical software was used for statistical processing, and the measurement data were represented by $\bar{x}\pm s$. One-way analysis of variance (ANOVA) was used for multiple group comparisons, and the Bonferroni-corrected *t*-test was adopted for pairwise comparisons between groups. In addition, *P* < 0.05 indicated that the difference was statistically substantial.

## 3. Results

### 3.1. MRI Findings in Patients with Cerebral Infarction

**Figure 2:**
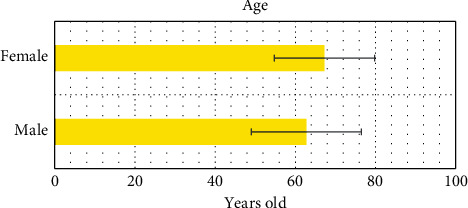
Comparison on age between men and women included in the study.

Figure 2 reveals the comparison results on age between men and women included in the study.

**Figure 3:**
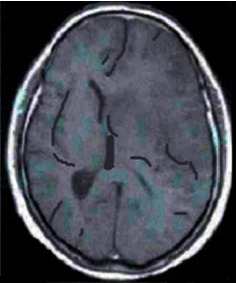
T1 MRI image of a patient with cerebral infarction eight hours after the onset.

**Figure 4:**
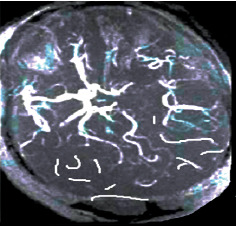
T2 MRI image of a patient with cerebral infarction eight hours after the onset.

**Figure 5:**
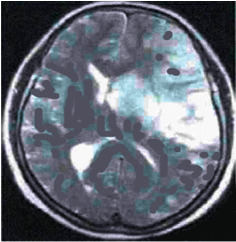
Enhanced T1 MRI image of a patient with cerebral infarction eight hours after the onset.

The images of the lesion in patient with cerebral infarction after MRI detection in Figures 3–5 show the following: right cerebral infarction was present, the brain sulcus disappeared, the midline shifted to the right, and the main left cerebral artery was occlusive.

### 3.2. Algorithm Segmentation Results

**Figure 6:**
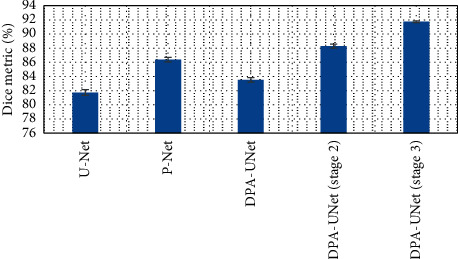
Comparison of the segmentation results between DPA-UNet and other methods.

By quantitative comparison of the segmentation results between DPA-UNet and other methods, it was found that the segmentation Dice coefficient of U-Net without additional supervision was 81.74 ± 0.40%, and the Dice coefficient of P-Net was 86.39 ± 0.31%. DPA-UNet was 83.52 ± 0.31% in the first stage, 88.29 ± 0.27% in the second stage, and 91.74 ± 0.12% in the third stage. There was no significant difference between each set of data, as shown in Figure 6. The larger the Dice coefficient was, the more accurate the segmentation was.

### 3.3. T1-Weighted Imaging Analysis

**Figure 7:**
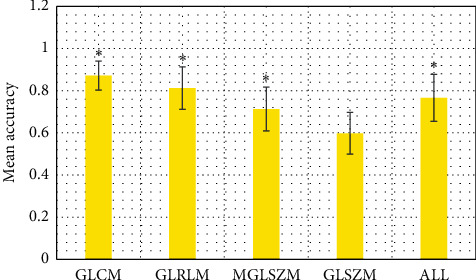
Analysis on T1-weighted images. ^*∗*^The difference was statistically marked compared with GLSZM (*P* < 0.05).

Figure 7 clearly expresses the average prediction accuracy and variance obtained by learning from the CNN and computing by computer. The best GLCM was 0.872 ± 0.069; the result of ALL was 0.766 ± 0.112; the result of GLRLM was 0.812 ± 0.101; the result of MGLSZM was 0.713 ± 0.104; the result of GLSZM was 0.598 ± 0.099. From these data, GLSZM was quite different from other data.

### 3.4. Enhanced T1-Weighted Imaging Analysis

**Figure 8:**
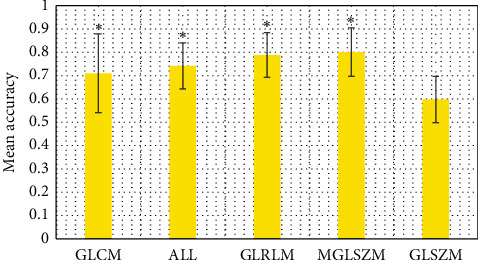
Analysis on enhanced T1-weighted images. ^*∗*^The difference was statistically marked compared with GLSZM (*P* < 0.05).

Figure 8 obviously indicates the average prediction accuracy and variance obtained through the CNN learning and computer calculation. Therefore, the best GLCM, ALL, GLRLM, MGLSZM, and GLSZM were 0.710 ± 0.169, 0.742 ± 0.099, 0.778 ± 0.096, 0.801 ± 0.104, and 0.598 ± 0.099, respectively. Based on the previously mentioned, GLSZM was quite different from other data.

### 3.5. T2-Weighted Imaging Analysis

**Figure 9:**
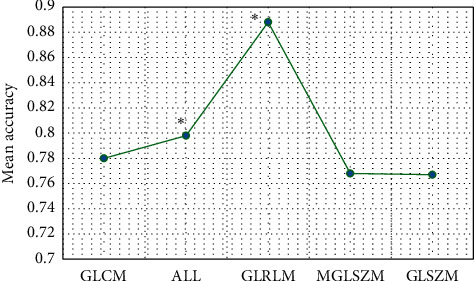
Analysis on T2-weighted images. ^*∗*^The difference was statistically marked compared with GLSZM (*P* < 0.05).

The average prediction accuracy and variance were obtained through the CNN learning and computer calculation, and the results are presented in Figure 9. The best GLCM, ALL, GLRLM, MGLSZM, and GLSZM were 0.780 ± 0.096, 0.798 ± 0.087, 0.888 ± 0.086, 0.768 ± 0.112, and 0.767 ± 0.100, respectively, suggesting that the GLSZM was quite different from other data.

## 4. Discussion

Cerebral infarction is the leading cause of death in China, and what is even more frightening is that the death toll of patients with cerebral infarction can account for almost 30% of the death toll due to disease. Moreover, the disease mostly occurs in the elderly, which greatly increases the risk of surgery when patients receive treatment, and the treatment costs are also very expensive [5]. What is more, the most important and independent risk factor for cerebral infarction is one of the most common chronic diseases, hypertension. Hypertension can change the pressure in blood vessels, stimulate the proliferation of smooth muscle cells, lead to atherosclerosis in arteries, or cause arterial vascular stenosis and insufficient local oxygen supply. At least half of the 50 patients in this study suffered from hypertension, with different severity and MRI partial maps of cerebral infarction. The mechanisms of cerebral infarction in China mainly include the following points: (1) Blood clotting is produced in the blood vessels of the brain; (2) nonbacterial endocarditis emboli fall off; (3) both. This means that vascular blood is particularly critical in the clinical treatment and diagnosis of cerebral infarction. However, it was not included in this study. In fact, blood vessels will not cause errors in visual judgment although they are very important [6–8]. Therefore, this research still focused on brain MRI. By performing brain MRI detection on these 50 patients and observing their image conditions, the main characteristics of the onset of cerebral infarction patients could be clearly and intuitively found.

In this study, it was found that feature description was one of the advantages of the judgment of cerebral infarction through the T1-weighted image analysis of MRI. This meant that artificial intelligence could improve performance to a considerable extent after learning and training. The human brain has a sophisticated structure and is extremely difficult to judge visually [9, 10]. Therefore, the features collected by CNN through MRI images would have considerable errors even after learning and training. In addition, a large amount of clinical data was still needed to supplement the clinical information, so that CNN could supplement the clinical information. MRI, CT, contrast imaging, nuclear medicine, and other imaging methods could also be used in this study. MRI could accurately detect the patient's condition of brain lesions in each time period through the change of signal [11, 12]. Thus, it could also be determined by the change of MRI T1 and T2 image time within eight hours of the onset of the patient's disease even if cerebral infarction was acute.

At this stage, almost all examinations for critically ill patients with cerebral infarction are through manual identification, and there are obvious subjective judgments, which cannot fully guarantee the accuracy of diagnosis [13, 14]. In this study, the deep learning-based CNN was used for the judgment of cerebral infarction. Generally speaking, the lesions in the brain of patients were usually manifested as decreased gyri density, blurred cerebral artery density, shallow cerebral sulcus and blurred gray and white matter, and so on within 12 hours after the onset of large-scale cerebral infarction. Due to edema in brain cells, sulcus might be even disappeared in the MRI image, and there was also the occurrence of cerebral island cortical edge ambiguity and other circumstances. Patients with cerebral infarction usually had an urgent onset, and MRI images were also more sensitive to scans of lesions in the brain. Therefore, with the help of deep learning-based CNN, cerebral infarction patients could even be scanned under the condition that the onset time was no more than six hours, and the state process and progress of cerebral infarction patients in the development of the disease could be shown through the change of T1 and T2 image time.

Based on the comparison of the best and worst results of different feature analysis for different MRI sequences, the GLCM, MGLSZM, and combination of ALL features obtained the best results on T1-weighted, T1C-weighted, and T2-weighted sequence images, respectively. Second, the modal results were all the worst. GLSZM was not suitable for T1-weighted and T2-weighted MRI to distinguish brain cancer subtypes, and the last classification results were obtained by GLCM in T1C-weighted image analysis. In addition, the combination of these features yielded some improvement in T2-weighted MRI analysis, but this did not mean that more features were better. This was because the combination of these features was not optimal for the other two imaging sequences. It should be noted that the results of the current experiment did not exceed 0.80 regardless of the mode or feature used. This meant that feature analysis might give physicians very limited diagnostic information in distinguishing cerebral infarction. For different MRI sequences, based on the area under the curve (AUC) pairs analyzed by different features, the combination of all features could make the final AUC obtain the best results. Generally speaking, the predictive performance of any feature description was off by about 10%, suggesting that the stability of the system was further improved by spatial comparison of different modes, and T2-weighted MRI showed certain advantages in differentiating the two types of brain tumors. The CNN with deep learning and MRI results can be used to obtain this result. MRI assisted by deep learning-based CNN can clearly display the focal part of patients with cerebral infarction in the early stage of the disease. If the T1 and T2 images of MRI are prolonged, the edema of the focal part of brain tissue can be observed more obviously in patients with cerebral infarction. In the process of artificial intelligence-assisted diagnosis, the brain tissue edema in patients with cerebral infarction can be found to further develop into vasogenic edema in the case of the occurrence and progression of the disease in patients with cerebral infarction, and the amount of protein exudation in the brain lesion further increases. The results of this study revealed that, with the assistance of deep learning-based CNN, MRI was very sensitive to patients with cerebral infarction, and the diagnostic accuracy was also greatly improved.

The different imaging sequences of MRI (T1-weighted imaging, enhanced T1-weighted imaging, and T2-weighted imaging) were adopted based on the artificial intelligence assisted system of CNN. By comparing different MRI sequences, it was found that it was even difficult for computers to analyze significant differences in MRI images of cerebral infarction. In the process of extracting image features, if there were not enough sample data, CNN would produce larger images for the diagnosis of cerebral infarction, which required clinicians to have enough experience. The characteristics extracted by CNN from MRI images of patients with cerebral infarction could not be quantified in terms of essential attributes. Therefore, in subsequent studies, it is necessary to extract more traits or combine a variety of traits for analysis and judgment, so as to make timely and effective clinical diagnosis of cerebral infarction patients [15–18].

Studies have shown that when a physician applies CT imaging to examine a patient with a cerebral infarction, it usually takes 24 hours to show the results and the patient needs to be reexamined in time [19]. In the examination of patients with hemorrhagic cerebral infarction and ischemic cerebral infarction, it has been found that there is a high likelihood that there will be no different outcome in these two conditions if CT is used. In other words, it is likely to be manifested as cerebral gyratory sign, floating cloud sign, false tumor signs, expend hematoma type cerebral infarction, hematoma type cerebral infarction, and other results. The results of this study disclosed that, with the assistance of deep learning CNN, MRI had a very high sensitivity to patients with cerebral infarction, and the diagnostic accuracy was also greatly improved.

## 5. Conclusion

In this study, through the CNN-based MRI image characteristics analysis of cerebral infarction in critically ill patients, the image diagnosis method was found, which could reduce the subjective visual judgment error to a certain extent. It was proved that the introduction of deep supervision mechanism could enhance the recognition ability of U-Net, which was of great significance for the accurate extraction and reconstruction of MRI images of patients with cerebral infarction. The results of this research hoped to provide certain help and ideas for the clinical diagnosis of cerebral infarction in the development of CNN in imaging. The combination of medical image discipline and deep learning will be the main direction of future discipline development. At the same time, it should also be noted that there is no one general algorithm that can solve a certain kind of problems in medical images, so it is necessary to try more to combine different network models in the future to better solve problems. In practical clinical applications, the ability of CNN feature abstraction is not unreasonable. However, it is impossible to provide complete data optimization for clinical use because the MRI data of patients with cerebral infarction is not complete. In terms of imaging, it is extremely difficult to complete such a project. Therefore, this study hoped to establish an independent medical image evaluation system in subsequent supplementary research to eliminate the subjective visual errors of equipment and instruments or imaging physicians.

## Data Availability

No data were used to support this study.
